# Enterohepatic circulation of glucuronide metabolites of drugs in dog

**DOI:** 10.1002/prp2.502

**Published:** 2019-07-04

**Authors:** Xin Zhou, Kenneth C. Cassidy, Loyd Hudson, Michael A. Mohutsky, Geri A. Sawada, Junliang Hao

**Affiliations:** ^1^ Drug Disposition Lilly Research Laboratories Indianapolis Indiana; ^2^ Medicinal Chemistry Lilly Research Laboratories Indianapolis Indiana

**Keywords:** enterohepatic circulation, glucuronidation, microbiome hydrolysis, P‐glycoprotein

## Abstract

The enterohepatic circulation (EHC) of drugs is often the result of the direct glucuronidation, excretion of the metabolite into bile, followed by hydrolysis to the aglycone by the gut microbiome and finally reabsorption of drug into the systemic circulation. The aim of present study to identify key factors in determining the EHC in dog for canagliflozin and DPTQ, two compounds cleared by UDP‐glucuronosyltransferase (UGT) mediated O‐alkyl glucuronidation and cytochrome P450 (P450) mediated oxidation. The pharmacokinetic profiles of the drugs were compared between bile duct cannulated (BDC) and intact beagle dogs after a single intravenous administration. A long terminal elimination phase was observed for DPTQ but not for canagliflozin in intact dogs, while this long terminal half‐life was not seen in BDC animals, suggesting the EHC of DPTQ. Quantification of parent drugs and glucuronide metabolites in bile, urine and feces indicated low recovery of parent in bile and urine and low recovery of conjugated metabolites in urine for both drugs, while biliary excretion of these glucuronide metabolites in BDC dog were low for canagliflozin but much higher for DPTQ. The increased fecal recovery of parent drug in intact dog and the lack of glucuronide metabolites suggested the hydrolysis of DPTQ‐glucuronides by gut microbiome. Subsequent characterization of in vitro hepatic metabolism and permeability properties indicated the hepatic fraction metabolized by UGT, hydrolysis of metabolites, and reabsorption of the aglycone were key factors in determining the EHC of DPTQ.

**Abbreviations**
ABT: 1‐Aminobenzotriazole
AUC: area under the concentration‐time curve
AUC_inf_: area under the concentration‐time curve from time zero extrapolated to infinite
BDC: bile duct cannulated
BSA: bovine serum albumin
Cl: clearance
Cl_int_: intrinsic clearance
Cl_int,u_: unbound intrinsic clearance
DPTQ: 2‐(2,6‐dichlorophenyl)‐1‐((1S,3R)‐5‐(2‐hydroxy‐2‐methylpropyl)‐3‐(hydroxymethyl)‐1‐methyl‐3,4‐dihydroisoquinolin‐2(1H)‐yl)ethan‐1‐one
EDTA: ethylenediaminetetraacetic acid
EHC: enterohepatic circulation
fm: fraction of metabolism
HPLC: high‐performance liquid chromatography
MRP: multidrug resistance‐associated protein NER, net efflux ratio
MS: mass spectrometer
P450: cytochrome P450
Papp: apparent permeability coefficients
PD: pharmacodynamics
P‐gp: P‐glycoprotein
PK: pharmacokinetics
*t*_1/2_: terminal half‐life
UDPGA: uridine 5’‐diphosphateglucuronic acid
UGT: UDP‐glucuronosyltransferases
*V*_dss_: volume of distribution at steady state

## 1 INTRODUCTION

Enterohepatic circulation EHC) is a process composed of a circuit of hepatic metabolism, biliary excretion, gut microbiome metabolism, followed by reabsorption from the gut back to systemic circulation.^1^, ^2^ Forty‐five drugs were identified that undergo EHC in a recent review article.^3^ Among these, the drug itself may be directly secreted into bile without undergoing metabolism; others undergo conjugation in the liver, such as glucuronidation and sulfation, then the metabolites are excreted in bile, stored in gallbladder, and released to the gut, where they undergo hydrolysis back to the parent drug by gut microbiome.^2^ Pharmacokinetic parameters such as half‐life (*t*_1/2_), volume of distribution (*V*_dss_), area under the concentration‐time curve AUC) and bioavailability often are substantially affected by EHC with orally administered drugs,^2^ demonstrating the importance of assessing the magnitude of EHC to model the pharmacokinetic/pharmacodynamics PK/PD) for a clinical candidate in drug discovery. This is especially true for compounds where a minimum concentration needs to maintained over a 24 hours period. A recent study investigated the fraction of hepatic metabolism fm) in dogs and suggested EHC will also affect the cumulative fm estimation of drugs that are metabolized by both P450s and UGTs in the liver, when the glucuronide metabolites are excreted into bile and hydrolyzed by gut microbiome during the process of EHC.^4^

In the present study, the EHC of canagliflozin, known to be cleared by P450 mediated oxidation and UGT mediated O‐glucuronidation in human,^5^, ^6^ and DPTQ 2‐2,6‐dichlorophenyl)‐1‐1S,3R)‐5‐2‐hydroxy‐2‐methylpropyl)‐3‐hydroxymethyl)‐1‐methyl‐3,4 dihydroisoquinolin‐21H)‐yl)ethan‐1‐one,^7^ a discovery project compound primarily cleared by hepatic metabolism of UGT and P450, were investigated in dog. The dog was used since it is a preclinical species that is more physiologically relevant to human than rodents with respect to bile secretion,^8^ biliary drug excretion 9 and colonic absorption.^10^ The PK profiles were compared between bile duct cannulated (BDC) and intact dogs after a single intravenous administration of the drugs, and the distribution of O‐glucuronide metabolites in plasma, bile, urine and feces was quantified to explore the fate of the metabolites during EHC. Subsequent in vitro hepatic metabolism and permeability studies were performed to characterize the physicochemical and metabolic properties that favor EHC. Given the disposition of canagliflozin has been reported for humans,^11^ a comparison of the in vitro and in vivo metabolic and excretion data of canagliflozin in human to those obtained with dog was performed in order to identify the species differences in the process of EHC.

## 2 MATERIALS AND METHODS

### 2.1 Chemicals and reagents

**Figure 1:**
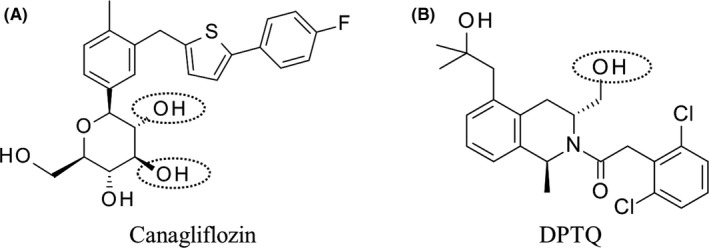
Chemical structures of drugs and sites of glucuronidation

Canagliflozin, (1S)‐1,5‐anhydro‐1‐C‐(3‐{[5‐(4‐fluorophenyl)thiophen‐2‐yl]methyl]}‐4‐methylphenyl)‐D‐glucitol, DPTQ, 2‐(2,6‐dichlorophenyl)‐1‐((1S,3R)‐5‐(2‐hydroxy‐2‐methylpropyl)‐3‐(hydroxymethyl)‐1‐methyl‐3,4‐dihydroisoquinolin‐2(1H)‐yl)ethan‐1‐one and the O‐glucuronide metabolite of DPTQ (Figure 1) were synthesized at Lilly Research Laboratories (Indianapolis, IN). 1‐Aminobenzotriazole (ABT), uridine 5’‐diphosphoglucuronic acid (UDPGA) and β‐glucuronidase Type H‐2 from *Helix pomatia* were purchased from Sigma Aldrich (St. Louis, MO). Solvents (water, methanol and acetonitrile) were of high‐performance liquid chromatography (HPLC) grade (Fisher Scientific, Pittsburg, PA).

### 2.2 Animals

Male beagle dogs were obtained from Charles River Laboratories Inc (Wilmington, MA). Pharmacokinetic (PK) studies in intact dogs were conducted at Covance (Greenfield, IN), while bile duct cannulation surgery and PK studies were conducted in these dogs at Covance (Madison, WI). Dogs (weight: 9‐12 kg), were acclimated prior to study initiation according to Standard Operating Procedures (Covance). All intravenous compound administration was by an intravenous catheter implanted in the cephalic vein. Access to food and water was allowed ad libitum. All animal procedures were approved by the Institutional Animal Care and Use Committee at Covance. Replacement bile was not administered during the study since collection of bile was scheduled for a period of 48 hours, replacement is typically administered for studies lasting more than 60 hours.

### 2.3 In vivo pharmacokinetics and excretion studies

Canagliflozin and DPTQ, both at 0.5 mg/kg, were administered intravenously in 25 mmol/L phosphate buffer (pH 8) containing 10% dimethylacetamide (v/v), 15% ethanol (v/v) via a cephalic vein. Total volume administered was 1 mL/kg. Dogs were housed in stainless steel metabolic cages during the study. Serial blood samples were collected from the jugular vein at 0.08, 0.25, 0.5, 1, 2, 4, 8, 12, 24, 36 and 48‐hours post‐dose with collection tubes containing anticoagulant K3‐EDTA. Plasma was obtained by centrifugation at 1600*g* for 10 minutes. Cumulative urine samples were collected between 0‐12, 12‐24, 24‐36 and 36‐48 hours and feces were collected between 0‐24 and 24‐48 hours in both intact and BDC dogs. Bile samples were collected between 0‐12, 12‐24, 24‐36, 36‐48 hours in BDC dogs. The weights of all samples except blood and plasma were recorded. All the samples were stored at −70°C until analysis by liquid chromatography tandem‐mass spectrometry (LC‐MS/MS) was performed.

### 2.4 Determination of pharmacokinetic parameters

Noncompartmental pharmacokinetic parameters were calculated using Watson version 7.4 (Thermo Scientific, Waltham, MA). Statistical significance of differences for pharmacokinetic parameters between two groups was examined with Student's *t* test using Sigmaplot 12.5 (Systat Software Inc, San Jose, CA).

### 2.5 Determination of fraction of CYP‐mediated oxidative metabolism in hepatocytes

The intrinsic clearance was determined in cryopreserved dog and human hepatocytes essentially as described previously.^4^ The assay incubation contained 0.3 µmol/L compound and hepatocytes at a concentration of 10^6^ cells/mL, with and without 30 minutes pre‐incubation of 1 mmol/L ABT. Intrinsic clearance measurement was initiated by the addition of compound and the incubation quenched at 0.6, 15, 30, 60 and 90 minutes for DPTQ, and at 0.6, 15, 30, 60, 120 and 240 minutes by addition of acetonitrile with 0.1% formic acid. Samples were centrifuged at 3500 *g* for 10 minutes and the supernatant was analyzed to quantify parent compound loss and the intrinsic clearance values (μL/min/10^6^ cells) were calculated using following equation for incubations with and without ABT.

$${\text{CL}}_{\text{int}}=-k_{\text{dep}}\times \text{incubation volume}/10^{6}\text{cells}$$

In which the depletion rate constant (*k*_dep_) is the slope determined using linear regression from the log transformed percentage remaining on the *y*‐axis vs time on the *x*‐axis (min^−^^1^). The fraction of metabolism (fm) by P450 was determined by the measurement of the percentage of inhibition of Cl_int_ by ABT.

### 2.6 Hepatocyte metabolite profile

Cryopreserved dog and human hepatocytes were obtained from BioreclamationIVT (Baltimore, MD). Incubations were performed in a CO_2_ incubator at 37°C using a 24‐well plate containing 250 000 cells/well with and without 1 mmol/L ABT pre‐incubation for 30 minutes. A stock solution of canagliflozin and DTPQ was added to medium to give a final incubation concentration of 2 μmol/L. The incubations including the media and cells were quenched after 4 hours with an equal volume of acetonitrile containing 200 ng/mL niflumic acid as internal standard. Samples were centrifuged at 3500 *g* for 10 minutes and the supernatant was analyzed by liquid chromatography coupled to a diode array UV detection and mass spectrometry system consisting of the Acquity ultra‐performance liquid chromatography column, Synapt G2‐S mass spectrometer, and an ultra‐performance liquid chromatography photodiode array detector (Waters, Milford, MA). The chromatographic separation of metabolites was achieved on a Waters 1.7‐μm Acquity BEH C18 (100 × 2.1 mm) column maintained at 60°C. The mobile phase consisted of solvent A (0.2% formic acid in water for DPTQ and 10 mmol/L ammonium acetate in water for canagliflozin) and solvent B (100% acetonitrile). The samples were eluted, at a flow rate of 0.5 mL/min with a gradient for 5 minutes. The LC/MS full scan data were processed by Metabolynx (Waters, Milford, MA) to identify metabolites. Product ion spectra were acquired to confirm the metabolites. The mass spectrometer was operated in positive ion mode with an electrospray ionization source. The parameters for the chamber were: capillary 1.0 kV, sample cone 50 V, source temperature 120°C, desolvation 500°C, and collision energy 25 V. The peak areas for parent, metabolite and internal standard were integrated to allow for comparison between conditions.

### 2.7 Bi‐directional transport across MDCK cells

MDCKII cells stably expressing human wild‐type P‐gp were obtained from the Netherlands Cancer Institute Amsterdam, The Netherlands). MDCK cells were maintained and the assay was conducted essentially as described previously.^12^, ^13^ Transport was measured in both directions across uninhibited and inhibited cell monolayers using a substrate concentration of 5 μmol/L diluted from a 10 mmol/L DMSO stock solution final DMSO concentration of 0.05%) and a single 60‐min time interval. 2.5 μmol/L LSN335984 was used to selectively inhibit P‐gp.^12^ The apparent permeability coefficients (Papp) were estimated as the slope of the mass transported per 60 minutes relative to the total recovered mass according to.^14^ The basal‐to‐apical (B‐A)/apical‐to‐basal (A‐B) Papp ratios were calculated in the absence or presence of inhibitor; dividing the uninhibited Papp B‐A/A‐B) ratio by the inhibited Papp B‐A/A‐B) ratio yields the net efflux ratio NER). If NER is >3.0, then the compound is classified as a P‐gp substrate.^12^

### 2.8 Hydrolysis of glucuronide metabolites in plasma, urine, bile and feces by β‐glucuronidase

Aliquots of plasma, urine, bile or feces homogenate (50 µL) were incubated with equal volume of 0.2 mol/L sodium acetate buffer (pH 5.0), and 15 μL of β‐glucuronidase (>85 000 units/mL) in a 37°C water bath overnight. The samples were extracted for LC‐MS/MS after the hydrolysis was performed. The disappearance of the glucuronide metabolite peak in the metabolite profile with β‐glucuronidase in different matrices suggested the glucuronide metabolite was hydrolyzed successfully. The concentration of O‐glucuronides was determined by subtracting the concentration from the aliquot without treatment of β‐glucuronidase from the one with enzyme treatment.

### 2.9 Liver and kidney microsomal glucuronidation assay

Intrinsic clearance of the compounds by glucuronidation was conducted in human and dog liver and kidney microsomes. Microsomes (0.5 mg/mL) with or without 2% bovine serum albumin (BSA), 100 mmol/L Tris‐HCl buffer, (pH 7.5), containing 5 mmol/L MgCl_2_ and 50 µg of alamethicin were mixed and placed on ice for 15 minutes. Canagliflozin or DPTQ (2 µmol/L final concentration) were added, and the mixture was pre‐incubated at 37°C for 3 minutes. A 0‐minute time point was obtained and the reaction initiated by the addition of UDPGA (5 mmol/L final concentration). Aliquots of the incubation (50 µL) were removed at various time points 5, 15, 30, 60 and 120 minutes, and added to the quench solution (1:1 acetonitrile:water). The zero minute time points were also quenched in the same manner. Substrate concentrations were then measured by LC/MS and the intrinsic clearance values (μL/min/mg protein) were calculated using following equation.

$${\text{CL}}_{\text{int}}=-k_{\text{dep}}\times \text{incubation volume}/\text{mg protein}$$

In which the depletion rate constant (*k*_dep_) is the slope determined using linear regression from the log transformed % remaining on the *y*‐axis vs time on the *x*‐axis (min^−^^1^). The lower limit of quantification of intrinsic clearance was 1.8 µL/min/mg protein. To determine the unbound intrinsic clearances the microsomal Cl_int_ values were divided by the respective microsomal fraction unbound values. The microsomal unbound fraction both with and without 2% BSA was determined as described previously.^15^

### 2.10 LC‐MS/MS quantification of drugs and metabolites

Canagliflozin and DPTQ in plasma, bile, urine and feces were quantified using LC‐MS/MS. All samples plasma, urine and bile) were mixed with an organic internal standard solution essentially described previously,^15^ to precipitate protein and centrifuged, and the resulting supernatants were analyzed using AB Sciex API 4000 quadrupole mass spectrometer (Applied Biosystems, Foster City, CA) equipped with a TurboIonSpray interface. The feces samples were homogenized in MeOH:H_2_O (1:1) and shaken vigorously to obtain slurry. The homogenates then went through the sample extraction along with other matrices. The blank plasma, urine, bile and feces was spiked with internal standard for calibration curve. The pumps were Shimadzu LC‐10AD units with a SCL‐10A controller (Kyoto, Japan), and a Z‐215 liquid handler (Gilson, Middleton, WI) was used as the autosampler. All compounds were chromatographically separated using a Betasil javelin C18 20 × 2.1‐mm 5‐μm HPLC column (Thermo Fisher Scientific, Waltham, MA). The mobile phase consisted of solvent A (1 mmol/L ammonium bicarbonate in water) and solvent B (1 mmol/L ammonium bicarbonate in methanol). Drugs and metabolites were detected in positive ion mode, using selected ion mode: canagliflozin 462.2 → 191.1 *m/z*, DPTQ 436.2 → 158.9 *m/z*, and DPTQ‐O‐glucuronide 612.2 → 418.1.

## 3 RESULTS

### 3.1 Pharmacokinetics of drugs in intact and bile duct cannulated (BDC) dog

**Figure 2:**
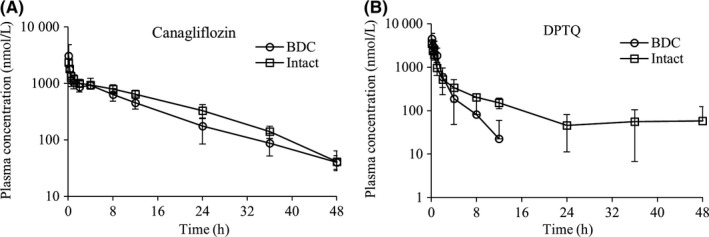
Plasma concentration of (A) canagliflozin and (B) DPTQ in beagle dog after a single intravenous administration at 0.5 mg/kg in bile duct cannulated (BDC) and intact dog. Data represent means ± SD (n = 4)

Physicochemical properties molecular weight (MW), clogP and pKa (Table 1) of canagliflozin and DPTQ were calculated using tools supplied by ChemAxon (http://www.chemaxon.com). Topological polar surface areas TPSA) were calculated based on the method described previously.^16^ The number of hydrogen bond donors (HBD) was calculated as the sum of NH and OH atoms and hydrogen bond acceptors (HBA) as the sum of N and O. Both canagliflozin and DPTQ are neutral and lipophilic compounds, while canagliflozin displays higher number of HBD/HBA and higher TPSA than DPTQ. The structures of the two drugs and sites of glucuronidation are presented in Figure 1. Both are cleared mainly by metabolism. For the metabolism by conjugation in humans, canagliflozin is primarily glucuronidated into two inactive metabolites by UGT1A9 and UGT2B4. DPTQ is metabolized by mixed oxidation and direct glucuronidation in human hepatocyte; the authentic standard of the O‐glucuronide was synthesized at Eli Lilly and the structure was confirmed using nuclear magnetic resonance spectroscopy (NMR, data not shown). The pharmacokinetic (PK) profile and parameters of the two drugs after intravenous administration at 0.5 mg/kg were compared between intact and BDC dogs (Figure 2 and Table 2). The plasma clearance was obtained by dividing the dose by area under the concentration‐time curve from time zero extrapolated to infinity (AUC_0‐∞_) or to 48 hours (AUC_0‐48_ hours for DPTQ in intact dogs) from noncompartmental analysis. The results showed that clearances of canagliflozin (1.32 and 0.93 mL/min/kg in BDC and intact dog respectively) and DPTQ (4.43 and 2.73 mL/min/kg in BDC and intact dog respectively) from plasma were much lower than dog hepatic blood flow (*Q*h = 30.9 mL/min/kg^17^). No statistically significant differences were observed for clearances of canagliflozin or DPTQ between intact and BDC dogs. However, the terminal half‐life (*t*_1/2_) of DPTQ was significantly longer in intact dog compared with the BDC dog, accompanied by the flat and tailing elimination phase and increased volume of distribution at steady state (*V*_dss_) (Figure 2 and Table 2), suggesting the enterohepatic circulation (EHC).

**Table 1:** Physicochemical properties of drugs

Compound	MW	clogP	pKa		Number of HBA/HBD	TPSA
			Most acidic	Most basic		
Canagliflozin	445	3.52	12.6	−2.98	5/4	90
DPTQ	436	4.2	14.9	−1.87	4/2	61

**Table 2:** Plasma pharmacokinetic parameters for canagliflozin and DPTQ in bile‐duct cannulated and intact dog

Parameters		Canagliflozin		DPTQ		DPTQ‐O‐glucuronide
		BDC	Intact	BDC	Intact^b^	Intact
AUC_0‐48 hr_	µmol/L*hr	15.7 ± 5.4	20.7 ± 3.7	5.89 ± 3.3	7.94 ± 2.5	2.61 ± 1.5
AUC_inf_	µmol/L*hr	16.4 ± 5.9	21.2 ± 3.9	5.89 ± 3.3	NA	2.6 ± 1.7
*t*_1/2_	hr	10.9 ± 1.8	8.05 ± 0.62	2.50 ± 2.3	NA	
*V*_dss_	L/kg	0.87 ± 0.28	0.72 ± 0.13	0.33 ± 0.07^a^	1.48 ± 0.25	
CL	mL/min/kg	1.32 ± 0.50	0.93 ± 0.18	4.43 ± 3.1	2.73 ± 1.2	

Note

Dogs were given canagliflozin and DPTQ at 0.5 mg/kg by IV bolus. Values were derived from plots shown in Figure 2. Results shown represent mean ± SD from four animals.

^a^ *P* < 0.01 compared to the parameters in intact animals.

^b^ The elimination phase was flat and AUC_inf_ was not extrapolated, the CL and *V*_dss_ was CL_0‐48 h_ and *V*_dss 0‐48 hr_

### 3.2 Recovery of parent drugs and glucuronides in urine, feces and bile of intact and BDC dogs

The extent of renal excretion of canagliflozin, DPTQ and canagliflozin‐glucuronides was negligible (<1% of dose) and the renal recovery of DPTQ‐glucuronide was low (<5% of dose). The biliary excretion of parent drugs represented 4.66 and 8.91% of dose for canagliflozin and DPTQ respectively, indicating the metabolism was primarily responsible for clearance of both drugs in dog (Table 3). The biliary excretion of glucuronide metabolites represented 15% of dose for canagliflozin and 35.8% of dose for DPTQ in BDC dog, whereas the fecal recovery of glucuronide metabolites was 0.37% for canagliflozin and 0.80% for DPTQ in intact dog during 48 hours, suggesting the hydrolysis of the glucuronides after biliary excretion in the gut. The significantly increased fecal recovery of the parent drug of DPTQ in intact as compared to BDC dog was also consistent with the hydrolysis of DPTQ‐O‐glucuronides by gut microbiome and excretion to feces (Table 3).

**Table 3:** Recovery of parent and glucuronide metabolite for canagliflozin and DPTQ in bile, urine and feces in bile‐duct cannulated (BDC) and intact dog

Drug	Animal	% dose in urine		% dose in bile		% dose in feces	
		Parent	O‐gluc	Parent	O‐gluc	Parent	O‐gluc
Canagliflozin	BDC	0.05 ± 0.08	0.11 ± 0.08	4.66 ± 1.4	15.0 ± 0.9	3.3 ± 0.7	ND
	Intact	0.27 ± 0.15	0.08 ± 0.10	NA	NA	2.25 ± 1.3	0.37 ± 0.32
DPTQ	BDC	ND	3.69 ± 5.7	8.91 ± 8.9	35.8 ± 22.9	2.48 ± 4.8	ND
	Intact	0.64 ± 1.1	1.44 ± 0.70	NA	NA	13.5 ± 5.8^a^	0.80 ± 1.2

Note

Dogs were given canagliflozin and DPTQ at 0.5 mg/kg by IV bolus. The bile, urine and feces samples were collected in BDC dogs, and the urine and feces was collected in intact dog over 48 hours post‐dose. The bile, urine and feces samples after canagliflozin and DPTQ administration were separated into two aliquots, one for bioanalytical assay of parent drugs (1), and one treated with β‐glucuronidase (2) and analyzed for parent drugs as described in Materials and Methods. The concentration of O‐glucuronides was determined by subtracting the concentration of (1) from (2). Results shown represent means ± SD (n = 4).

Abbreviations: NA, bile concentration in intact dog was not available; ND, the concentration was below the quantitative limit 1 ng/mL.

a *P* < 0.05 compared to that in BDC dog.

### 3.3 In vitro hepatocyte Cl_int_ and metabolite profile

The intrinsic clearances (Cl_int_) of canagliflozin and DPTQ characterized by parent loss in dog hepatocytes were inhibited 12.7 and 46.5% by ABT, respectively (Table 4), The percentage of the metabolism of DPTQ not inhibited by ABT (53.5%) in dog hepatocyte was similar to the percentage of dose excreted in bile as DPTQ‐glucuronide over 48 hours (35.8 ± 22.9%). However, the percentage of metabolism of canagliflozin not inhibited by ABT (87.3%) was much higher than percentage of dose excreted in bile as canagliflozin‐glucuronide. The human hepatocyte Cl_int_ was inhibited to the similar extent by ABT as dog for canagliflozin, while minimal inhibition (%) was observed for DPTQ in human hepatocytes (Table 4).

**Table 4:** Hepatocyte Cl_int_ of canagliflozin and DPTQ in the presence and absence of 1‐Aminobenzotriazole (ABT)

Drug	Species	Cl_int_ without ABT	Cl_int_ with ABT	% of inhibition by ABT
		µL/min/10^6^ cells		
Canagliflozin	Dog	6.5 ± 3.6	6.0 ± 3.3	12.7 ± 14.1
	Human	12.7 ± 2.0	10.1 ± 1.4	20.1 ± 5.1
DPTQ	Dog	38.6 ± 8.5	18.3 ± 5.9	46.5 ± 6.6
	Human	5.8 ± 2.0	5.5 ± 2.2	7.8 ± 9.6

Note

Incubation contained 0.3 µmol/L compound and 10^6^/mL dog and human hepatocytes with and without pre‐incubation of 1 mmol/L ABT for 30 minutes, as described in Materials and Methods. Results shown represent means ± SD (n = 4 or 5).

**Figure 3:**
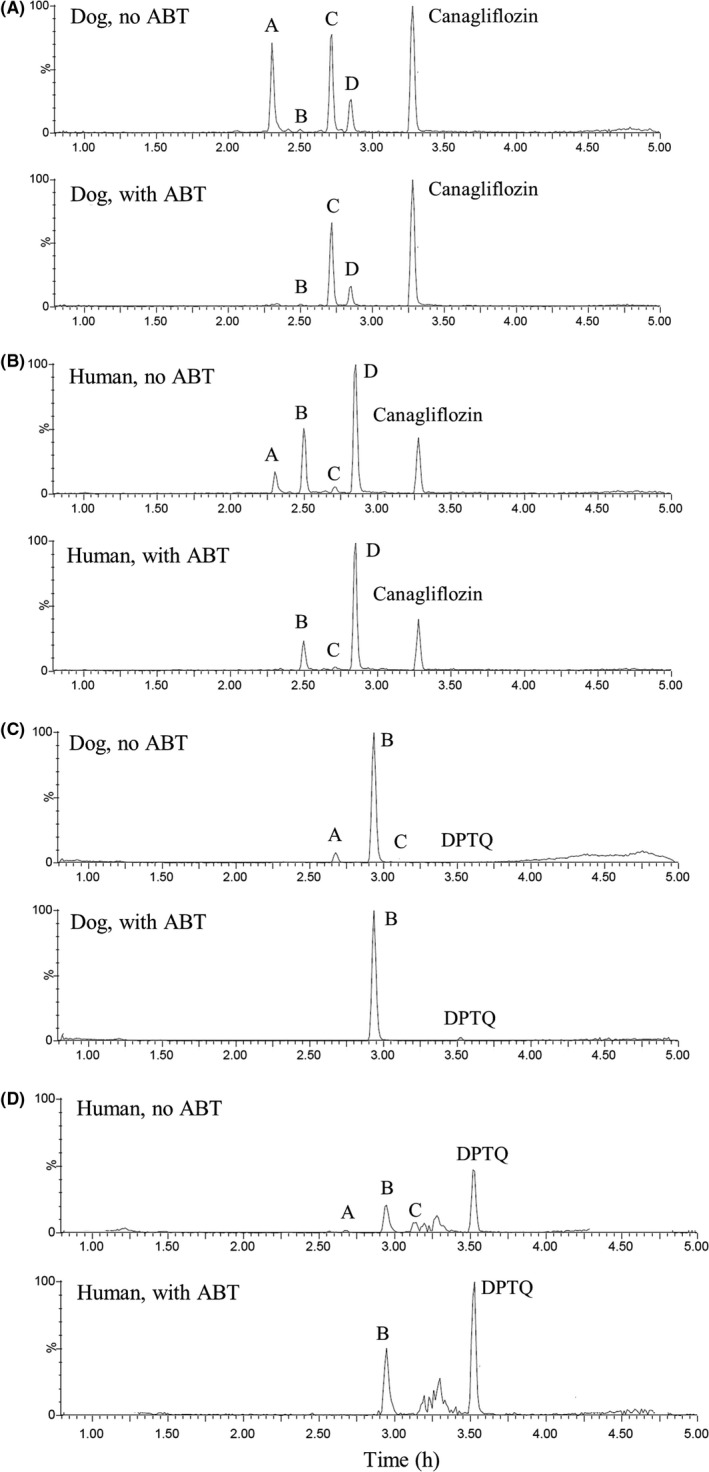
Metabolite identification of canagliflozin (A) and (B), and DPTQ (C) and (D) in dog and human hepatocytes in the presence and absence of ABT. For canagliflozin, metabolite A, P+O+sulfate; B, P+glucuronide; C, P+O‐2H; D, P+glucuronide. For DPTQ, metabolite A, P+O+glucuronide; B, P+glucuronide; C, P+O

The subsequent dog hepatocyte metabolite profile indicated that oxidation and glucuronidation were major metabolic pathways for DPTQ in dog hepatocytes (Figure 3 and Table 5). In the presence of ABT, the primary oxidative metabolite C was not detected, whereas glucuronide metabolite metabolite B) peak in the profile was not meaningfully reduced <20% change for metabolite/internal standard peak area ratio). Product A P+O+glucuronidation) was a secondary metabolite and the formation was inhibited by ABT. The metabolite profile for DPTQ in human hepatocytes was similar to that observed with dog. For canagliflozin, the metabolites B and D were the two direct glucuronidation products in dog hepatocytes, reported as M5 and M7 reported previously.^5^ The secondary metabolite A (P+O+sulfation) is likely formed by oxidation followed by sulfation according to the inhibition of the product formation in the presence of ABT and the fact that no direct sulfate conjugate was observed. The metabolite C (P+O‐2H), the alcohol oxidation product, did not appear to be formed by P450 mediated oxidation, given the minimal inhibitory effect that was observed in the presence of ABT. It could be formed by alcohol dehydrogenase and aldehyde dehydrogenase in hepatocytes. The high abundance of metabolite C in dog hepatocytes could explain the discrepancy between the low recovery of canagliflozin‐glucucuronide in vivo and low percentage of metabolism inhibited by ABT in vitro. All four major metabolites detected in dog hepatocytes were also formed in human hepatocytes; however, the relative abundance of metabolite C was much lower in human hepatocytes, consistent with previous report that O‐glucuronidation was the primary pathway in the metabolic clearance of canagliflozin.^5^, ^6^

**Table 5:** Peak area of metabolite in the presence and absence of ABT in hepatocyte metabolite identification

Compound	Species	Metabolite	Tentative Metabolite Identification	*m/z*	RT (min)	Peak area (‐ABT)	Peak area (+ABT)
Canagliflozin	Dog		Parent (P)	443	3.28	7808	8448
		A	P+O+sulfate	539	2.30	4929	ND
		B	P+glucuronide	619	2.50	147	90
		C	P+O‐2H	457	2.71	5683	4715
		D	P+glucuronide	619	2.85	2125	1151
		IS		283	2.59	8707	6696
	Human		Parent (P)	443	3.28	3414	2019
		A	P+O+sulfate	539	2.30	1346	ND
		B	P+glucuronide	619	2.50	3807	1121
		C	P+O‐2H	457	2.71	472	162
		D	P+glucuronide	619	2.85	8427	4866
		IS		283	2.59	8771	5517
DPTQ	Dog		Parent (P)	436	3.52	33	244
		A	P+O+glucuronide	628	2.68	724	ND
		B	P+glucuronide	612	2.95	10 225	8916
		C	P+O	452	3.13	42	ND
		IS		283	3.51	722	661
	Human		Parent (P)	436	3.52	2810	2364
		A	P+O+glucuronide	628	2.68	84	ND
		B	P+glucuronide	612	2.95	1305	1148
		C	P+O	452	3.13	393	ND
		IS		283	3.51	493	506

Note

Hepatocyte metabolite identification was conducted with and without pre‐incubation of 1 mmol/L ABT as described in Materials and Methods. The peak area values were derived from plots in Figure 3.

Abbreviations: P, parent drug; IS, internal standard; RT, retention time; ND, not detected.

### 3.4 Liver and kidney microsomal glucuronidation

The unbound intrinsic clearances (Cl_int,u_) determined by parent loss in liver and kidney microsomes were examined in order to understand whether the O‐glucuronides detected in urine are produced by glucuronidation in the kidney or are formed in the liver, excreted into blood, and then eliminated from circulation by the kidney. The microsomal incubation was conducted with or without 2% bovine serum albumin (BSA), which is known to reduce *K*_m_ values for substrates of UGT isoforms that are inhibited by long‐chain unsaturated fatty acids released by incubation with human liver microsomes.^18^ The Cl_int,u_ was higher in the presence of BSA in human microsomes for canagliflozin (liver and kidney) and DPTQ (liver), as well as in dog liver microsomes for DPTQ (Table 6). The substantial Cl_int,u_ of glucuronidation in human kidney suggested the canagliflozin O‐glucuronides detected in human urine could be locally produced in the kidney (Table 6). The negligible glucuronidation in both liver and kidney microsomes of dog was consistent with negligible circulating and urinary recovery of canagliflozin‐O‐glucuronides. In contrast, the glucuronidation activity towards DPTQ was abundant in dog liver microsomes but not detected in kidney microsomes, suggested the urinary recovery of DPTQ‐O‐glucuronide in dog is mainly from circulation after hepatic metabolism. The lower glucuronidation activity in human liver microsomes was consistent with lower hepatocyte Cl_int_ compared to dog for DPTQ, and glucuronidation intrinsic clearance by human kidney microsomes was not detected (Table 6).

**Table 6:** Hepatic and kidney UGT Cl_int_ and unbound Cl_int_ of canagliflozin and DPTQ

Drugs	Species	BSA	Liver microsomes	Kidney microsomes
			Cl_int_ and (Cl_int,u_)	
			µL/min/mg protein	
Canagliflozin	Dog	−	ND	ND
		+	ND	ND
	Human	−	ND	ND
		+	7.6 (422)	4.0 (222)
DPTQ	Dog	−	99 (123)	ND
		+	79.4 (667)	ND
	Human	−	ND	ND
		+	5.6 (47.1)	ND

Note

Incubation contained 2 µmol/L compound and 0.5 mg/mL liver and kidney microsomes with and without 2% BSA, as described in Materials and Methods. The unbound Cl_int_ (in the parenthesis) was calculated using Cl_int_ divided by microsomal unbound fraction. Results shown represent average of two independent experiment.

Abbreviations: ND, Cl_int_ < 1.8 µL/min/mg protein, the quantitative limit.

### 3.5 MDCK permeability and gut reabsorption

Passive membrane permeability was high for DPTQ and moderate for canagliflozin (Table 7). DPTQ was not a P‐gp substrate, whereas canagliflozin was determined to be a P‐gp substrate in a P‐gp‐transfected MDCKII cells in the absence or presence of 2.5 µmol/L P‐gp specific inhibitor LSN335984, with net efflux ratio (NER) >3 (Table 7). Compounds with relatively high numbers of hydrogen bond donors and acceptors as well as higher polar surface area are often P‐gp substrates,^12^ as is the case with canagliflozin (Table 1). This also agrees with a previous report that canagliflozin is a P‐gp substrate.^6^ The differences in the permeability and P‐gp liability are likely to determine the extent of reabsorption to portal circulation and subsequent EHC.

**Table 7:** In vitro permeability and net efflux ratio in P‐gp assay

Compound	Cell line	Papp_A‐B_ (10^–6^cm/s)	Papp_B‐A_ (10^–6^cm/s)	Papp_B‐A_/Papp_A‐B_	Net efflux ratio (Pgp substrate)
Canagliflozin	MDR1‐MDCKII_inhibitor	10.8 ± 1.4	14.1 ± 0.1	1.3	17.1 (S)
	MDR1‐MDCKII_control	4.0 ± 1.8	89.2 ± 8.2	22	
DPTQ	MDR1‐MDCKII_inhibitor	48.9 ± 5.9	53.5 ± 0.4	1.1	1.3 (NS)
	MDR1‐MDCKII_control	43.0 ± 4.5	62.6 ± 2.6	1.5	

Note

Incubation contained 5 µmol/L compound with and without 2.5 µmol/L P‐gp inhibitor, as described in Materials and Methods. Results shown represent means ± SD (n = 3).

Abbreviations: S, substrate; NS, non‐substrate.

## 4 DISCUSSION

**Figure 4:**
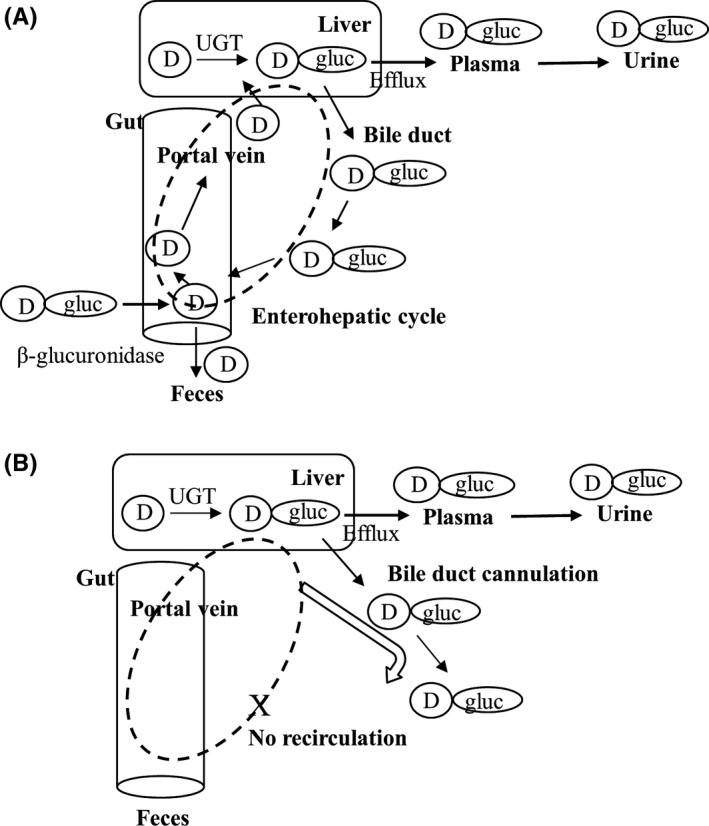
Formation of O‐glucuronides in the liver and distribution to plasma, urine, bile and feces in (A) intact and (B) BDC dog. D, drug, D‐gluc, drug glucuronide metabolite

Enterohepatic circulation (EHC) can increase apparent volume of distribution (*V*_dss_) and prolong half‐life (*t*_1/2_) of drugs. The more extensive the recycling, the more prolonged the *t*_1/2_ and the greater the volume increase.^19^ In the BDC dog, the EHC is blocked and the increase in apparent *V*_dss_ and prolonged *t*_1/2_ are no longer observed (Figure 4. Therefore, the differences in the PK profiles and the levels of O‐glucuronides in different matrices, from intact and BDC animals allowed the investigation of EHC. It is not clear if the bile duct cannulation would demonstrably affect hepatic metabolism and function of transporters in dog. In the present study of canagliflozin and DPTQ, the recoveries of parent drugs and the glucuronides in urine were not significantly different between intact and BDC dogs; the % dose of bile glucuronides were consistent with hepatic fraction of metabolism by UGT in in vitro hepatocyte intrinsic clearance assays, suggesting the minimal effects of bile duct cannulation on the metabolism and transport of the compounds. Although both drugs displayed low hepatic extraction and a similar clearance mechanism, the increased *V*_dss_ and prolonged *t*_1/2_, were observed for DPTQ in intact compared to BDC dog, but not for canagliflozin, triggering the subsequent investigation of underlying mechanisms and the identification of key physicochemical and metabolic properties in determining EHC. The human metabolism and disposition data was reported for canagliflozin,^5^ allowing the assessment of species differences between dog and human from a translational perspective (Table 8).

**Table 8:** Summary of in vitro assay and in vivo model to investigate O‐glucuronide enterohepatic circulation in preclinical setting

Drug	Species	Hepatic fm by UGT^a^	Biliary excretion % of glucuronide^b^	Urinary excretion % of glucuronide^c^	MDCK permeability (P‐gp substrate)	EHC
Canagliflozin	Dog	Low	Low	Low		No
	Human	High		Moderate^d^	Moderate (yes)	No
DPTQ	Dog	Moderate	Moderate	Low		Yes
	Human	High			High (no)	

a Low 0%‐30%, moderate 30%‐70%, high 70%‐100% in hepatocytes.

b Low 0%‐30%, moderate 30%‐70%, high 70%‐100% of dose from bile duct cannulated (BDC) dog.

c Low 0%‐30%, moderate 30%‐70%, high 70%‐100% of dose from BDC and/or intact dog, source of glucuronides could be confirmed by circulating glucuronides level and in vitro kidney glucuronidation.

d From human ^14^C study.

The EHC of canagliflozin in human was also reported to be negligible.^11^ However, the underlying mechanism resulting in the same outcome was distinct between dog and human. Glucuronidation represented the major hepatic metabolic pathways in human for canagliflozin, whereas oxidation is primarily responsible for the hepatic metabolism in dog as indicated in previous publications^5^, ^6^ and the present study. Although the hepatic Cl_int_ of canagliflozin was not substantially inhibited by ABT in dog, the formation of the carboxylic acid metabolite by dehydrogenase relative to glucuronides, as revealed by hepatocyte metabolite profile along with the low biliary recovery of O‐glucuronides in BDC dog, gives further evidence of a low fraction of hepatic glucuronidation. The AUC of circulating glucuronides M5 and M7 in total was ~½ of canagliflozin AUC in human after oral administration of canagliflozin at 188 mg, whereas the plasma concentration of glucuronides were not detected in dog after oral administration at 4 mg/kg^5^ or after intravenous administration at 0.5 mg/kg in the present study. The observation could be due to relatively lower fraction of metabolism by UGT and less formation of glucuronides in dog liver. Alternatively, it may also indicate the potential differences in the hepatic efflux of O‐glucuronides to the plasma between dog and human, presumably due to the species differences in the expression and/or activity of multidrug resistance‐associated protein (MRP) on the sinusoidal (basolateral) side of the liver.^20^ The urinary recovery of M5 and M7 in total accounted for 31.9% of dose in human,^5^ whereas the urinary recovery of these glucuronides was ~0.1% of dose in both BDC and intact dog. While the difference could be explained by the species differences in the kidney glucuronidation between human and dog in the present study and/or potential species difference in the hepatic efflux described above,^20^ much evidence does point to the difference in liver glucuronidation.

In contrast, the circulating AUC of DPTQ‐glucuronides was 33% of AUC of DPTQ in dog, with the resulting 1.4%‐3.7% urinary recovery of O‐glucuronides of DPTQ due to efflux of glucuronide metabolites formed in the liver, given the kidney glucuronidation of DPTQ was negligible and much lower than that in the liver. Evidence shows that the EHC for DPTQ was not extensive since there was little impact on AUC and clearance between intact and BDC dogs. It is due to the low hepatic extraction of DPTQ, since the magnitude of EHC is associated with hepatic extraction ratio; a higher extraction ratio was shown to cause more extensive EHC,^21^ whereas DPTQ displayed very low hepatic extraction.

Given the complexity in the EHC, the PK profile and parameters were less variable for canagliflozin in both animal groups and DPTQ in the BDC dog compared to DPTQ in intact dog. The dog is generally considered as the most biologically relevant preclinical species in investigating biliary excretion of xenobiotics.^8^, ^9^ In addition, the dog model is considered a suitable surrogate for the estimation of human colonic absorption of passively absorbed drugs.^10^ Gaps still exist, however, with respect to species differences in the hepatic efflux of glucuronides and the resulting biliary and urinary distribution of O‐glucuronides, as well as with the potential difference in hydrolysis of O‐glucuronides by gut microbiomes of dogs and humans.^2^ The β‐glucuronidase activities in microbiome of human gut were reported along the gut wall from proximal to distal regions as 0.02 to 0.9 µmol of substrate degraded/hr/g content, resulting in the higher efficiency in hydrolysis in the distal regions eg, cecum and colon,.^2^ A preliminary study incubating 10 µmol/L of DPTQ‐O‐glucuronide with human feces under anaerobic conditions resulted in 100% hydrolysis of the glucuronide within 24 hours (unpublished data). Recent publications of human ^14^C studies of drugs with diverse structures that all undergo UGT mediated glucuronidation as major clearance pathways, all displayed low recovery of O‐glucuronides in fecal samples, indicating a high efficiency of hydrolysis by human gut microbiome.^22^, ^23^, ^24^ The low recovery of O‐glucuronides in the feces of dog in the present study and human in the previous study^5^ demonstrate the efficient hydrolysis of canagliflozin‐O‐glucuronides in both species. As β‐glucuronidase activities in microbiome of human gut increases from proximal to distal regions, the relative P‐gp expression P‐gp/villin integrated optical density ratio) also progressively increases,^25^ which may have a more profound effect on the reabsorption of a P‐gp substrate. Therefore, the substantial recovery of parent drug in feces and lack of evidence for enterohepatic circulation in human^5^ is likely the combined effects of the moderate permeability and P‐gp substrate properties for canagliflozin. In contrast, the high fm by UGT in human hepatocytes as well as high permeability and lack of P‐gp activity on the compound, would lead to a greater extent of EHC of DPTQ via O‐glucuronide formation and hydrolysis. The relative contribution of hepatic efflux in the urinary and biliary distribution of DPTQ‐O‐glucuronide in human however, is not known. All of those factors would lead to uncertainties in the prediction of PK/PD in clinical trials.

It should be noted that the drugs were intravenously administered in fed condition in the present study, a simplified model to focus on identification of metabolic and physicochemical properties that influence EHC. Both canagliflozin 6 and DPTQ exhibited negligible intestinal glucuronidation in human intestinal microsomes data not shown). Extensive intestinal glucuronidation could complicate the assessment of fecal recovery of drugs and glucuronides for both intravenously and orally administered drug, such as ezetimibe,^26^ but this was not the case with the two compounds in this study. In addition, the investigation of EHC was focused on O‐alkyl‐glucuronides in the present study, and EHC may not be as extensive in drugs that are metabolized to N‐glucuronides or acyl‐glucuronides, given the hydrolysis of N‐glucuronides by β‐glucuronidase by gut microbiome may not be as effective as for O‐glucuronides^27^; acyl‐glucuronides could also undergo rearrangement and produce β‐glucuronidase resistant species^28^ that would complicate the assessment of EHC.

In conclusion, we demonstrated the hepatic UGT metabolism and/or permeability are crucial to EHC using canagliflozin and DPTQ as tool molecules. The models and the assays utilized in the present study, as summarized in Figure 4 and Table 8, could facilitate the preclinical assessment and clinical prediction of EHC qualitatively and/or semi‐quantitatively, for drugs that are cleared via glucuronidation and sulfation and excreted in the bile. In drug candidate selection at the preclinical to clinical development interface, dog and human in vitro data along with in vivo data from dog, may help in refining human PK predictions for compounds that undergo EHC.

## DISCLOSURES

None declared.

## AUTHOR CONTRIBUTIONS

Participated in research design: Zhou, Cassidy, Mohutsky. Conducted experiments: Cassidy, Hudson, Sawada. Contributed new reagents or analytic tools: Cassidy, Hao. Performed data analysis: Zhou, Hudson, Cassidy. Wrote or contributed to the writing of the manuscript: Zhou, Cassidy, Mohutsky, Hao.
